# Association between Blood Parameters of Nutritional Status and Functional Status in Extreme Longevity

**DOI:** 10.3390/nu16081141

**Published:** 2024-04-12

**Authors:** Malgorzata Kupisz-Urbanska, Ewa Marcinowska-Suchowierska, Piotr Jankowski

**Affiliations:** 1 Department of Geriatrics, Medical Centre of Postgraduate Education, 01-813 Warsaw, Poland; 2 Department of Internal Medicine and Geriatric Cardiology, Medical Centre of Postgraduate Education, 01-813 Warsaw, Poland; emarcinowska1@gmail.com (E.M.-S.); piotrjankowski.eu@gmail.com (P.J.); 3 Department of Geriatrics and Gerontology, School of Public Health, Medical Centre of Postgraduate Education, 01-813 Warsaw, Poland; 4 Department of Epidemiology and Health Promotion, School of Public Health, Centre of Postgraduate Medical Education, 01-813 Warsaw, Poland

**Keywords:** longevity, longlived, nutrition components, proteins, nutritional status, health status, mobility, geriatric syndrome, malnutrition

## Abstract

Background: The relationship between functional and nutritional status in the geriatric population remains an issue of debate and there is a gap in the knowledge regarding this field in long-lived individuals. Aim: The main aim of this study was to assess the association between selected blood parameters of nutritional status and functional status in extreme longevity. Methods: The inclusion criteria were centenarians above 100 years of age who were examined at their homes, and blood samples were collected. The study group consisted of 170 individuals (25 men and 145 women, median age 100.75 years [100.29–101.58]). Results: Total protein and albumin serum concentration was significantly lower in long-lived individuals with severe functional decline compared to individuals with preserved functional status, *p* = 0.000001 and *p* = 0.0000, respectively. Iron serum level was significantly higher in the group with preserved functional status, *p* = 0.04. Preserved functional status was positively correlated with total protein serum concentration (*p* = 0.000), albumin concentration (*p* = 0.000), and iron serum level (*p* = 0.029). A negative correlation was stated between c-reactive protein (CRP) and functional status (*p* = 0.032). Univariable logistic regression analysis showed that the functional status of long-lived individuals depends on total protein (OR 2.89, CI 95% [1.67–5.0]) and albumin concentrations (OR 2.34, CI 95% [1.39–3.92]). Multivariable backward stepwise logistic regression analysis showed that a total protein concentration was the only variable independently related to the preserved functional status (OR 3.2, 95% Cl [1.8–5.67]). Conclusions: In long-lived individuals, the total serum protein and albumin levels are lower in centenarians with severe functional decline, and they correlate with functional status. Total protein serum concentration is the only factor independently related to the preserved functional status in extreme longevity.

## 1. Introduction

Life expectancy has nearly doubled over the last century and the number of long-lived persons has risen steadily and significantly all over the world [1]. Meanwhile, it is still an issue of debate whether old age is only a continuation of lifespan or rather a distinct stage. Moreover, with extreme old age, the health status perception seems to change, and mobility, ability, and self-sufficiency become the key values from the health perspective [2]. An extended lifespan is the main risk factor not only for developing diseases with high prevalence (such as cardiovascular diseases, cancer, or diabetes mellitus) but also is often characterized by specific geriatric symptoms like loss of mobility or malnutrition [3,4]. Physiological reduction in appetite contributes to food intake reduction. Researchers highlighted that persons over 80 years consume about 30% less energy per day in comparison to younger adults [5]. Inappropriate nutrition status contributes strongly to increased morbidity, functional decline, sarcopenic loss of independence, and mortality [6]. In the oldest old geriatric population, a vicious cycle of protein energy waiting, progressive frailty, and loss of physical performance accompanied by vitamin and micronutrient deficiencies become a challenge for the health care systems all over the world [7,8]. A strong statistically significant association was found between nutritional status and frailty syndrome. Individuals with malnutrition had a significantly higher risk of functional decline assessed by daily living activities, twice for instrumental activities of daily living (IADL) and three times for activities of daily living (ADL) compared to those without malnutrition [9].

The data from the literature highlight that most of the centenarians were independent in their daily living activities into their 90s. Despite the medical and socioeconomic heterogeneity in centenarians (including physical status, cognitive function, socioeconomic status, and cultural or historical background) they were described by Franceschi et al. as a model of successful aging three decades ago [10,11]. Concomitantly, high nutritional risk was associated with functional status decline and with predictors of negative clinical outcomes [12]. Analysis of blood biochemistry and routine examinations of the quantitative and morphological changes in blood cells provide not only the biological characteristics estimating the function of organs but also constitute blood-based biomarkers of aging [13,14,15]. Observational studies conducted with community-dwelling individuals, and institutionalized patients (including hospitals and long-term care institutions) highlighted a strong inverse correlation between the serum protein concentration and the risk of subsequent multimorbidity and mortality [16]. These findings suggest that albumin serum concentration could become a crucial prognostic indicator that can be applied in population-based studies. However, there is still a gap in the knowledge concerning a wide range of nutrient deficiencies and their relationship with functional status in longevity.

The main aim of the study was to assess the association between selected blood parameters of nutritional status and functional status in extreme longevity. 

## 2. Patients and Methods

This study utilized a multidisciplinary program of successful aging including all individuals over 100 years of age in Poland reported to the Universal Electronic System for Registration of the Population (PESEL number) database. We analyzed centenarians with enough collected blood samples to perform blood tests. The only exclusion criterion was lack of informed consent. The Bioethics Committee of the Central Clinical Hospital of the Ministry of the Interior and Administration in Warsaw approved the study and the centenarians’ multidisciplinary program (PBZ-KBN-022/PO5/1999 Genetic and environmental factors of longevity), or their relatives gave written informed consent for participation in the study.

After they were identified by the PESEL number, the centenarians were contacted by mail and invited to participate in the centenarians’ study. All long-lived persons were visited at their homes and blood samples were collected. The examinations of the centenarians were conducted throughout the year. The study group consisted of 170 individuals (25 men and 145 women, median age of 100.75 years). The methods included taking a medical history (interviews with individuals and/or the family), physical examination including height and body mass measurements, and collecting a blood sample. A medical and sociodemographic survey form was tested during the preliminary stage of the centenarians’ project. The questionnaire contained general sociodemographic information, lifestyle indicators, and detailed medical history that included questions concerning gender, date, place of birth, place of residence, and activities of daily living. Laboratory tests included serum levels of creatinine, glucose, total protein, albumin, c-reactive protein (CRP), calcium, magnesium, sodium, potassium, folic acid, cobalamin (vitamin B12), iron, ferritin, total cholesterol, and blood cell count. Blood samples were analyzed using standard biochemical and morphological tests, and routine biochemistry automated analyzers and automated cell counters were used, respectively. For everyone, the functional status was estimated. We assessed functional status by classifying every patient into one of the five lifestyle groups according to their main daily living activities: laying, laying sitting, sitting, sitting walking, or walking. Then we divided centenarians into two groups: the first with severe functional decline consisted of laying and laying-sitting individuals (SFD group, 58 individuals) and the second group with preserved functional status consisted of sitting, siting-walking, or walking individuals (PFS group 112 individuals). 

## 3. Statistical Analysis

Continuous variables are presented as medians (interquartile ranges [IQRs]), while categorical values are presented as percentages with 95% confidence intervals (CI) when appropriate. The Shapiro–Wilk test was used to assess the normality. To compare variables without normal distribution, we used the Mann–Whitney U test or the Kruskal–Wallis test, as appropriate. Univariable logistic analysis was performed to identify factors associated with functional status. Factors with a statistical significance of *p* < 0.1 in univariable analysis were included in a multivariate logistic regression analysis to identify predictors of preserved functional status. A *p*-value of less than 0.05 was considered statistically significant. Statistical analyses were performed using the statistical suite StatSoft. Inc. (Tulsa, OK, USA, 2017), STATISTICA (data analysis software system) version 13.0. www.statsoft.com (accessed on 28 March 2024).

## 4. Results

Baseline characteristics of study participants are presented in Table 1. The female/male coefficient in centenarians was 5.6, and the majority (over 70%) of examined individuals lived in cities. The median body mass index (BMI) was less than 23 kg/m^2^, and over the last 50 years of life, the median weight loss was 12.24 kg and the median height loss was 13.5 cm.

Less than a half of the participants—69 individuals (40.5%) took any vitamin or electrolyte supplements, 37 (22%) took multivitamin supplements, 10 (6%) took vitamin B supplements, 22 (13%) took other supplements (including vitamin C and E preparations and multicomponent preparations containing electrolytes). All supplemented preparations contained low or very low doses of vitamins, and oral nutritional supplements were not used permanently by any of the participants. 

A comparison of the characteristics of laboratory parameters (median, first, and third quartile) in women and men is presented in Table 2. Significant differences were found between women and men in total protein concentration *p* = 0.0456, ferritin serum concentration *p* = 0.0070, and creatinine *p* = 0.0000. 

The median values of laboratory parameters and the comparison in groups with different functional statuses are presented in Table 3. Total protein and albumin serum concentration was slightly below the recommended levels in long-lived individuals with severe functional decline and was in the normal range in individuals with preserved functional status. Significant differences were found between groups in total protein and albumin concentration, *p* = 0.000001 and *p* = 0.000, respectively. Iron levels were in the normal range in both groups with significantly higher levels in groups with preserved functional status, *p* = 0.044.

The median concentration of vitamin B12 was in the normal range, with low values in the 1st and 3rd quartile, without significant differences between SFD and PFS groups, *p* = 0.4206. The median concentration of folic acid was higher in groups with preserved functional status without statistical significance between groups, *p* = 0.1181. In all study groups, the main biochemical markers of liver and kidney function were stable, without significant differences between the severe functional decline group, and the group with preserved functional status, for aspartate aminotransferase *p* = 0.8704 and creatinine *p* = 0.4406, respectively. Statistically significant differences were not stated between groups when comparing electrolytes (sodium and potassium serum concentration was in the upper normal limit in both groups), blood morphotic parameters, inflammatory biochemical markers, glucose levels, and cholesterol concentrations. Glucose and cholesterol serum concentrations were in the normal range in both groups with slightly lower values of cholesterol in the SFD group, without statistical significance, *p* = 0.0917. A total cholesterol concentration below 150 mg/dL was found in 23 individuals in the study group (60% of them were centenarians with preserved functional status).

Significant correlations were found between total protein, albumin serum concentration, vitamins, cholesterol, iron, and hemoglobin. Preserved functional status was positively correlated with total protein serum concentration, albumin concentration, and iron serum level. A negative correlation was stated between c-reactive protein and functional status, Table 4.

Univariable logistic regression analysis of nutritional status blood parameters showed that functional status of long-lived individuals (laying, laying sitting, sitting, sitting walking, or walking) is related to total protein concentration (OR 2.89, CI 95% [1.67–5.0])) and albumin concentration (OR 2.34, CI 95% [1.39–3.92]). Multivariable backward stepwise logistic regression analysis was applied in models containing nutritional status and blood parameters as predictors of preserved functional status. From all estimated blood parameters, we found the total protein concentration was the only variable independently related to preserved functional status (OR 3.2, 95% Cl [1.8–5.67]).

## 5. Discussion

From a medical point of view, the centenarians are a remarkable and exceptional population because they not only avoided extended physiological decline and age-related diseases, but the rate of processes was slow enough to respond to minor stresses of daily life and avoid their accumulation. We believe this is the first study to focus on biochemical markers of nutrition and correlations with functional status in extreme old age. Considering that our study group demonstrated distinctive characteristics of extreme aging, we envisage that it is likely to be representative of the entire population of long-lived persons. We observed reduced anthropometric measures in long-lived individuals. Body mass index value below the recommended level for older adults and the high index of weight and height loss (over the last 50 years of centenarians’ life) could be an equivalent of osteosarcopenic changes, the loss of muscle mass (sarcopenia) and the loss of bone mass (osteopenia or osteoporosis). These data are consistent with other studies. It is emphasized that with advancing age, the physiological process of muscle strength and function loss results in a poor quality of life and impaired mobility. Thereby, there is a need for secondary prevention in octogenarians and nonagenarians to prevent functional decline in extreme old age [17,18].

In our study, centenarians presented a good profile of lipids and glucose levels, a normal range of liver and kidney blood biochemical parameters that were consistent with data from the literature and could be due to a reduced number of cardiometabolic complications and to the effect of prolonged periods of healthy aging [19,20]. This study did not specify the differences between men and women when comparing lipids, glucose profiles, or blood cell count. Apart from creatinine serum concentration, the only statistically significant differences between males and females were shown for total protein and ferritin. These findings conform with data from the literature concerning well-established gender distinction with age [21].

Interestingly, electrolyte serum concentrations were in the upper limit of the normal range. However, the data from the literature in this field are confounding. Lower sodium intake was associated with worse cognitive function in older adults in comparison to individuals with higher sodium intake. Authors of the study suggest that for the maintenance of cognitive function, older adults may be advised to avoid very low sodium intake [22]. On the other side, a higher daily sodium diet was independently correlated with lower successful aging characterized by high education level, physical and social activities, outdoor activities, and the total number of clinical cardiovascular risk factors [23]. Extreme old age homeostasis of serum biochemical parameters concentration probably becomes more complex than in younger adults. 

It has been shown in animal models that blood chemistry measures and complete blood count (CBC) profiles vary based on age. Researchers suggest that some of the changes like significant changes in albumin, lymphocytes, and hemoglobin are associated with a healthy aging process. It could be that exhibited values outside of this defined healthy aging reference will allow more accurate diagnostics and treatments in long-lived individuals [24,25]. 

Simultaneously, studies focused on biological aging (including blood chemistry quantifications) in humans suggest that the blood chemistry measures of biological aging may be predictive of disability and mortality in older adults [26]. 

Presumably, factors that play a crucial role in the processes of aging in younger groups do not have the same impact in older groups. Data on the longevity population in the older adults’ group are still lacking, although a positive correlation between protein and resistance training on physical performance was found [27].

Recently, Fanelli et al. revealed a relationship between nutrient intake, diet type, mortality, and functional limitations in 23,487 younger adults with diabetes mellitus. Authors highlighted that patients with a lower protein intake (<0.8 mg/kg), consumed reduced amounts of vitamins, including vitamin B12, and that they demonstrated a higher mean number of functional limitations [28]. Moreover, a high protein intake was correlated with decreased levels of overall mortality and cancer mortality in the population over 65 years [29,30]. As described in the literature, the amount of protein consumption should be adjusted to the characteristics and particularly the age of the subject and should consider long-lived individuals, metabolic disorders, and sarcopenia. However, the amount of protein in extremely advanced age is still an issue of debate [31].

Our data are consistent with other studies in younger geriatric populations. Schalk et al. in a prospective observation of Longitudinal Aging Study Amsterdam, indicated that chronic low serum albumin is a predictor of functional decline in older adults. Therefore, a decrease in serum albumin from normal to low levels but within the normal range was the strongest determinant of future decline in functional status. The authors suggest that a change in serum albumin concentration within the normal range measured between two points in time could be used in clinical practice as a general marker of future functional decline [32,33].

A Chinese study that aimed to investigate the nutritional risk, malnutrition, and nutritional support status of the oldest-old, hospitalized patients showed that nutritional risk was associated among others with functional decline which could contribute to a vicious cycle of malnutrition and functional decline [34]. 

The importance of albumin serum concentration was also underscored by authors of an observational study on sarcopenia and frailty. The study highlighted that among other markers, pre-operative albumin serum concentration was an independent factor for postoperative mortality within 90 days [35].

Lastly, a published meta-analysis concerning protein intake showed that high protein intake ameliorates physical status but not functional decline over time. The systematic review and meta-analysis of cross-sectional and longitudinal studies included 11,332 individuals with a mean age above 75 years. The authors concluded that protein intake higher than the recommended daily allowance was associated with better physical performance and greater muscle strength in older adults. However, the data concerning the high consumption of proteins and its impact on physical function decline over time are inconsistent [36,37]. A wide range of serum proteins increase with physiological aging. On that account, it remains to be the question of whether proteins’ normal ranges could be applied in age strata [38]. In our study, even though only albumin and total protein concentration were a predictive factor for preserved functional status, we found statistically significant correlations between proteins and folic acid, cholesterol, iron, and hemoglobin. That conforms with the findings of other studies that underscore the multifactorial aspect of nutritional status parameters [39,40].

We also found a negative correlation between c-reactive protein and functional status. Notwithstanding, in other studies the data are inconsistent. Li et al. did not find any correlation in older adults between inflammation markers and functional status. The authors suggest a probable role of multiple factors influence rather than one single marker [41]. However, in the Northern Manhattan Study of younger adults, higher serum CRP levels related to higher baseline disability, even when adjusting for baseline covariates, stroke, and myocardial infarction occurring during follow up [42].

Our study revealed that vitamin B12 status was suboptimal in all investigated individuals, but anemia was not found in centenarians and a dependence on functional status was not observed. It could be possible that with advanced age, the clinical impact of vitamin B12 depends more on diet diversity and other clinical factors. Liu et al. showed that the relationship between vitamin B12 serum concentration and the prevalence of anemia was significant only when the level of dietary diversity was relatively low [43]. 

In centenarians, our study found a positive correlation between iron and functional status. Several conditions contribute to iron deficiency, many of which are highly prevalent in the geriatric population. In an observational study, lower iron status was correlated not only with depressive mood, and cognitive function decline but also with the loss of mobility. These findings suggest that using iron as a continuous variable, rather than single measurements, may help find some associations with iron homeostasis and its clinical impact [44].

The role of the supplementation of vitamins remains an issue of debate. Notwithstanding that vitamin supplementation is supposed to increase physical performance and overall health status among older adults, it was not stated in a three-year longitudinal study. The Towards Useful Aging study highlighted that dietary supplement intake was associated neither with physical fitness nor cognitive function improvement. Our study showed that only 22% of centenarians used multivitamin low doses preparations. Therefore, the recommended vitamin and protein concentrations as well as supplementation particularly in long-lived patients are still under discussion [45].

Our study has some limitations. Considering the uniqueness of the study group it was impossible to provide observational prospective research. A limitation of our study was also the lack of extended data concerning anthropometric features and daily nutritional intake. Another limitation of our study was the number of participants living in the cities which could influence their prior physical activity and lifestyle. Despite the limitations mentioned above, our study shows the profile of the nutritional status of centenarians and the correlations with their functional status. Simultaneously, the study suggests the role of further studies in evaluating nutritional status and its clinical impact but also the need to establish optimal supplementation recommendations in extreme longevity.

The study group of centenarians seems to present a profile of prolonged healthy aging without strongly advanced deficiencies in blood-based biomarkers of aging, like cardio-metabolic serum profile and nutritional status. Notwithstanding the advanced aging processes, it becomes increasingly burdensome to separate the interdependent effects of physiological aging, nutritional status (including decreased food intake), and comorbidities that exert influence on the serum concentrations of proteins and other factors. For this reason, the periodic, regular assessment of serum nutritional biomarker concentrations could probably provide us with greater clinical value. Moreover, in clinical practice, complex nutritional serum parameters in conjunction with medical history data could be helpful in diagnosis and guiding treatment decisions, especially when considering the need for nutritional support. Undoubtedly, further studies of long-lived individuals are required to establish protocols for identifying vitamin, micronutrient, and protein deficiency symptoms and signs in clinical practice, as well as the optimal vitamin and protein status in extreme longevity. 

## 6. Conclusions

In long-lived individuals, the total serum protein and albumin levels are lower in centenarians with severe functional decline, and they correlate with functional status. Total protein serum concentration is the only factor independently related to the preserved functional status in extreme longevity.

## Figures and Tables

**Table 1:** Baseline characteristics of study participants.

Variable	Median [1st–3rd Quartile] or Number (%)
Sex	
Males	25 (15%)
Females	145 (85%)
Age, years,	100.75 [100.29–101.58]
Place of living, %	
City/town/village	70.7/12.9/16.4
Body mass, kg	50.56 [42.5–56.4]
Height, cm	149 [140.0–150.0]
Body mass index BMI, kg/m^2^	22.71 [20.42–26.62]
Body mass at the age of 50 years, kg	62.8 [56.5–64.0]
Height at the age of 50 years, cm	162.5 [155.4–163.0]

**Table 2:** Characteristics of laboratory parameters in women and men.

Laboratory Parameters (Units)	Women Median [1st–3rd Quartile]	Men Median [1st–3rd Quartile]	*p* Value
Vitamin B12 (pg/mL)	263.0 [208.0–352.0]	268.0 [215.0–334.0]	0.9060
Folic acid (ng/mL)	9.61 [5.2–16.25]	11.3 [7.17–20.00]	0.2236
Total protein (g/dL)	6.84 [6.38–7.32]	7.17 [6.65–7.5]	0.0456
Albumin (g/dL)	3.78 [3.46–3.08]	4.08 [3.69–4.25]	0.0939
Glucose (mg/dL)	84.5 [75.0–94.0]	82.0 [72.0–89.0]	0.2485
Cholesterol (mg/dL)	195.0 [168.0–228.0]	176.0 [147.0–216.0]	0.0732
Sodium (mmol/L)	142.0 [140.0–145.0]	143.0 [141.00–145.0]	0.3488
Potassium (mmol/L)	4.31 [4.0–4.78]	4.48 [3.75–4.7]	0.7900
Magnesium (mg/dL)	1.88 [1.73–2.15]	2.01 [1.84–2.23]	0.1713
Calcium (mg/dL)	8.88 [8.33–9.36]	8.89 [8.37–9.24]	0.8646
Iron (µg/dL)	67.0 [48.0–85.0]	73.0 [53.5–104.5]	0.1982
Ferritin ((µg/L)	83.31 [39.82–153.35]	158.28 [75.0–313.22]	0.0070
Creatinine (mg/dL)	0.93 [0.77–1.1]	1.2 [1.1–1.38]	0.0000
Aspartate aminotransferase (U/I)	18.0 [16.0–22.0]	20.0 [17.0–22.0]	0.1640
C-reactive protein (mg/dL)	5.43 [2.32–12.7]	7.37 [3.4–19.14]	0.2120
White blood cells (10^3^/μL)	5.73 [4.8–6.36]	5.95 [5.02–6.72]	0.3010
Lymphocytes (10^9^/L)	1.70 [1.28–2.12]	1.84 [1.19–2.01]	0.8977
Red blood cells (mln/μL)	4.07 [3.68–4.36]	4.13 [3.7–4.36]	0.6172
Hemoglobin (g/dL)	12.4 [11.3–13.3]	12.3 [11.4–13.5]	0.7330

**Table 3:** The median laboratory parameters values in the centenarians with severe functional decline (SFD group) and preserved functional status (PFS group).

Laboratory Parameters (Units)	Severe Functional Decline Median [1st–3rd Quartile]	Preserved Functional Status Median [1st–3rd Quartile]	*p* Value
Vitamin B12 (pg/mL)	287.0 [187.0–352.0]	258.5 [213.5–346.0]	0.4206
Folic acid (ng/mL)	7.92 [4.0–14.89]	10.39 [5.75–16.79]	0.1181
Total protein (g/dL)	6.49 [6.05–6.96]	7.05 [6.69–7.46]	0.000001
Albumin (g/dL)	3.49 [3.15–3.80]	3.94 [3.67–4.30]	0.0000
Glucose (mg/dL)	84.0 [78.0–93.0]	86.0 [74.00–95.00]	0.8762
Cholesterol (mg/dL)	181.0 [159.0–216.0]	200.5 [168.50–233.5]	0.0917
Sodium (mmol/L)	143.0 [139.0–145.0]	143.0 [141.00–145.0]	0.3632
Potassium (mmol/L)	4.4 [4.0–4.8]	4.3 [3.96–4.69]	0.3919
Magnesium (mg/dL)	1.85 [1.70–2.18]	1.96 [1.75–2.15]	0.6754
Calcium (mg/dL)	8.85 [8.06–9.18]	8.89 [8.51–9.38]	0.0923
Iron (µg/dL)	59.5 [38.0–82.0]	70.0 [55.0–87.0]	0.0444
Ferritin (µg/L)	100.67 [56.79–183.1]	83.65 [44.84–175.27]	0.6371
Creatinine (mg/dL)	0.97 [0.75–1.17]	0.96 [0.86–1.19]	0.4406
Aspartate aminotransferase (U/I)	18.0 [16.0–25.0]	19.0 [16.0–22.0]	0.8704
C-reactive protein, (mg/dL)	5.24 [2.80–23.51]	5.70 [2.31–10.16]	0.5247
White blood cells (10^3^/μL)	5.42 [4.76–6.5]	5.84 [4.84–6.39]	0.6522
Lymphocytes (10^9^/L)	1.70 [1.25–2.07]	1.70 [1.37–2.13]	0.5849
Red blood cells (mln/μL)	4.11 [3.54–4.34]	4.06 [3.69–4.39]	0.7120
Hemoglobin (g/dL)	12.4 [12.0–13.3]	12.4 [11.4–13.3]	0.9231

**Table 4:** Correlation between preserved functional status and blood quantitative biomarkers.

Variable	PFS	V B12	Ac F	Fer	TP	Alb	Glu	Chol	Fe	CRP	Lym
V B12	−0.130										
Ac F	0.098	0.067									
Ferr	0.051	0.100	0.249 *p* = 0.002								
TP	0.443 *p* = 0.000	−0.043	0.169 *p* = 0.040	0.149							
Alb	0.467 *p* = 0.000	0.050	0.303 *p* = 0.000	0.103	0.701 *p* = 0.000						
Glu	0.040	−0.022	−0.040	0.120	0.158	0.116					
Chol	0.093	0.027	0.004	−0.120	0.297 *p* = 0.000	0.405 *p* = 0.000	0.124				
Fe	0.180 *p* = 0.029	−0.035	0.038	0.116	0.278 *p* = 0.001	0.416 *p* = 0.000	0.231 *p* = 0.005	0.267 *p* = 0.001			
CRP	−0.177 *p* = 0.032	0.114	−0.080	0.143	−0.193 *p* = 0.019	−0.418 *p* = 0.000	0.042	−0.309 *p* = 0.000	−0.443 *p* = 0.000		
Lym	0.033	−0.122	−0.048	−0.079	0.134	0.048	0.094	0.042	0.013	−0.012	
Hgb	0.007	−0.128	−0.046	0.040	0.187 *p* = 0.024	0.254 *p* = 0.002	0.149	0.300 *p* = 0.000	0.512 *p* = 0.000	−0.353 *p* = 0.000	0.147

PFS—preserved functional status, V B12—vitamin B12, Ac F—folic acid, Fer—ferritin, TP—total protein, Alb—albumin, Glu—glucose, Chol—cholesterol, Fe—iron, CRP—C-reactive protein, Lym—lymphocytes, Hgb—hemoglobin.

## Data Availability

Data are contained within the article.

## References

[1] United Nations Department of Economic and Social Affairs, Population Division (2022). World Population Prospects 2022: Summary of Results.

[2] Franceschi C., Garagnani P., Morsiani C., Conte M., Santoro A., Grignolio A., Monti D., Capri M., Salvioli S. (2018). The Continuum of Aging and Age-Related Diseases: Common Mechanisms but Different Rates. Front. Med..

[3] Niccoli T., Partridge L. (2012). Ageing as a Risk Factor for Disease. Curr. Biol..

[4] Fries J.F. (1980). Aging, natural death, and the compression of morbidity. N. Engl. J. Med..

[5] Morley J.E., Silver A.J. (1988). Anorexia in the elderly. Neurobiol. Aging.

[6] Visvanathan R., Chapman I.M. (2009). Undernutrition and Anorexia in the Older Person. Gastroenterol. Clin. N. Am..

[7] Windahl K., Irving G.F., Almquist T., Lidén M.K., van de Luijtgaarden M., Chesnaye N.C., Voskamp P., Stenvinkel P., Klinger M., Szymczak M. (2018). Prevalence and Risk of Protein-Energy Wasting Assessed by Subjective Global Assessment in Older Adults with Advanced Chronic Kidney Disease: Results from the EQUAL Study. J. Ren. Nutr..

[8] Streicher M., van Zwienen-Pot J., Bardon L., Nagel G., Teh R., Meisinger C., Colombo M., Torbahn G., Kiesswetter E., Flechtner-Mors M. (2018). Determinants of Incident Malnutrition in Community-Dwelling Older Adults: A MaNuEL Multicohort Meta-Analysis. J. Am. Geriatr. Soc..

[9] Huynh N.T.H., Nguyen T.T.T., Pham H.K.T., Huynh N.T.H., Nguyen N.T., Cao N.T., Van Dung D. (2023). Malnutrition, Frailty, and Health-Related Quality of Life Among Rural Older Adults in Vietnam: A Cross-Sectional Study. Clin. Interv. Aging.

[10] Franceschi C., Monti D., Sansoni P., Cossarizza A. (1995). The immunology of exceptional individuals: The lesson of centenarians. Immunol. Today.

[11] Guay M., Dubois M.F., Corrada M., Lapointe-Garant M.-P., Kawas C. (2014). Exponential Increases in the Prevalence of Disability in the Oldest Old: A Canadian National Survey. Gerontology.

[12] Di Vincenzo O., Pagano E., Cervone M., Natale R., Morena A., Esposito A., Pasanisi F., Scalfi L. (2023). High Nutritional Risk Is Associated with Poor Functional Status and Prognostic Biomarkers in Stroke Patients at Admission to a Rehabilitation Unit. Nutrients.

[13] Mahlknecht U., Kaiser S. (2010). Age-related changes in peripheral blood counts in humans. Exp. Ther. Med..

[14] Blann A. (2014). Blood tests and age-related changes in older people. Nurs. Times.

[15] Bao H., Cao J., Chen M., Chen M., Chen W., Chen X., Chen Y., Chen Y., Chen Y., Aging Biomarker Consortium (2023). Biomarkers of aging. Sci. China Life Sci..

[16] Dennis H.S. (2021). What Do the Serum Proteins Tell Us about Our Elderly Patients?. J. Gerontol..

[17] Colón C.J., Molina-Vicenty I.L., Frontera-Rodríguez M., García-Ferré A., Rivera B.P., Cintrón-Vélez G., Frontera-Rodríguez S. (2018). Muscle and Bone Mass Loss in the Elderly Population: Advances in diagnosis and treatment. J. Biomed..

[18] Ono S., Sasabuchi Y., Yamana H., Yokota I., Okada A., Matsui H., Itai S., Yonenaga K., Tonosaki K., Watanabe R. (2024). Weight loss and functional decline in older Japanese people: A cohort study using large-scale claims data. Arch. Gerontol. Geriatr..

[19] Aliberti S.M., De Caro F., Funk R.H.W., Schiavo L., Gonnella J., Boccia G., Capunzo M. (2022). Extreme Longevity: Analysis of the Direct or Indirect Influence of Environmental Factors on Old, Nonagenarians, and Centenarians in Cilento, Italy. Int. J. Environ. Res. Public Health.

[20] Kupisz-Urbańska M., Jankowski P., Topór-Mądry R., Chudzik M., Gąsior M., Gil R., Gryka P., Kalarus Z., Kubica J., Legutko J. (2023). Survival in nonagenarians with acute myocardial infarction in 2014–2020: A nationwide analysis. Kardiol. Polska.

[21] Pitkanen H.T., Oja S.S., Kemppainen K., Seppa J.M., Mero A.A. (2003). Serum amino acid concentrations in aging men and women. Amino Acids.

[22] Rush T.M., Kritz-Silverstein D., Laughlin G.A., Fung T.T., Barrett-Connor E., McEvoy L.K. (2016). Association between dietary sodium intake and cognitive function in older adults. J. Nutr. Healthy Aging.

[23] Foscolou A., Critselis E., Tyrovolas S., Chrysohoou C., Naumovski N., Rallidis L., Polychronopoulos E., Matalas A.-L., Sidossis L.S., Panagiotakos D. (2020). The Association of Sodium Intake with Successful Aging, in 3349 Middle-aged and Older Adults: Results from the ATTICA and MEDIS Cross-sectional Epidemiological Studies. J. Nutr. Healthy Aging.

[24] Kupisz-Urbańska M., Stuss M., Kuryłowicz A., Jankowski P., Pilz S., Sewerynek E., Marcinowska-Suchowierska E. (2022). Fracture risk in obesity: A narrative review. Endokrynol. Polska.

[25] Hickmott A.J., Cervantes L., Arroyo J.P., Brasky K., Bene M., Salmon A.B., Phillips K.A., Ross C.N. (2023). Age-related changes in hematological biomarkers in common marmosets. Am. J. Primatol..

[26] Parker D.C., Bartlett B.N., Cohen H.J., Fillenbaum G., Huebner J.L., Kraus V.B., Pieper C., Belsky D.W. (2020). Association of Blood Chemistry Quantifications of Biological Aging with Disability and Mortality in Older Adults. J. Gerontol. Ser. A.

[27] Lee B.C., Kim K.I., Cho K.H., Moon C.-W. (2024). Effects of resistance training and nutritional support on osteosarcopenia in older, community-dwelling postmenopausal Korean females (ERTO-K study): A study protocol. BMC Geriatr..

[28] Fanelli S., Kelly O., Krok-Schoen J., Taylor C. (2021). Low Protein Intakes and Poor Diet Quality Associate with Functional Limitations in US Adults with Diabetes: A 2005–2016 NHANES Analysis. Nutrients.

[29] Simpson S.J., Le Couteur D., Raubenheimer D. (2015). Putting the Balance Back in Diet. Cell.

[30] Levine M.E., Suarez J.A., Brandhorst S., Balasubramanian P., Cheng C.-W., Madia F., Fontana L., Mirisola M.G., Guevara-Aguirre J., Wan J. (2014). Low Protein Intake Is Associated with a Major Reduction in IGF-1, Cancer, and Overall Mortality in the 65 and Younger but Not Older Population. Cell Metab..

[31] Iizuka K. (2021). Protein Amount, Quality, and Physical Activity. Nutrients.

[32] Kupisz-Urbańska M., Jankowski P. (2023). Patient with frailty syndrome undergoing cardiovascular interventions. Kardiol. Inwazyjna.

[33] Schalk B.W., Visser M., Penninx B.W., Baadenhuijsen H., Bouter L.M., Deeg D.J.H. (2005). Change in serum albumin and subsequent decline in functional status in older persons. Aging Clin. Exp. Res..

[34] Lai X., Zhu H., Du H., Huo X., Yu K. (2020). Nutritional status of Chinese oldest-old adults (≥80 years of age): A cross-sectional study in Beijing. Eur. J. Clin. Nutr..

[35] Guo K., Wang X., Lu X., Yang Y., Lu W., Wang S., Tang X., Wu Y., Xu Y., Chen Q. (2023). Effects of sarcopenia and frailty on postoperative recovery in elderly patients: A prospective cohort study. J. Cachex Sarcopenia Muscle.

[36] Coelho-Júnior H.J., Calvani R., Tosato M., Landi F., Picca A., Marzetti E. (2022). Protein intake and physical function in older adults: A systematic review and meta-analysis. Ageing Res Rev..

[37] Stoodley I.L., Williams L.M., Wood L.G. (2023). Effects of Plant-Based Protein Interventions, with and without an Exercise Component, on Body Composition, Strength and Physical Function in Older Adults: A Systematic Review and Meta-Analysis of Randomized Controlled Trials. Nutrients.

[38] Lind L., Sundström J., Larsson A., Lampa E., Ärnlöv J., Ingelsson E. (2019). Longitudinal effects of aging on plasma proteins levels in older adults—Associations with kidney function and hemoglobin levels. PLoS ONE.

[39] Chen B., Zhao H., Li M., Zhao T., Liao R., Lu J., Zou Y., Tu J., Teng X., Huang Y. (2024). Effect of multicomponent intervention on malnutrition in older adults: A multicenter randomized clinical trial. Clin. Nutr. ESPEN.

[40] Vialaret J., Vignon M., Hirtz C., Badiou S., Baptista G., Fichter L., Dupuy A.-M., Maceski A.M., Fayolle M., Brousse M. (2023). Use of dried blood spots for monitoring inflammatory and nutritional biomarkers in the elderly. Clin. Chem. Lab. Med. (CCLM).

[41] Li X., Ma L. (2024). From biological aging to functional decline: Insights into chronic inflammation and intrinsic capacity. Ageing Res. Rev..

[42] Dhamoon M.S., Cheung Y.-K., Moon Y.P., Wright C.B., Willey J.Z., Sacco R., Elkind M.S. (2016). C-reactive protein is associated with disability independently of vascular events: The Northern Manhattan Study. J. Am. Geriatr. Soc..

[43] Liu L., Zhou J., Chen C., Qu Y., Wang J., Lu F., Liu Y., Cai J., Ji S., Li Y. (2024). Vitamin B12 is associated negatively with anemia in older Chinese adults with a low dietary diversity level: Evidence from the Healthy Ageing and Biomarkers Cohort Study (HABCS). BMC Geriatr..

[44] Neidlein S., Wirth R., Pourhassan M. (2020). Iron deficiency, fatigue and muscle strength and function in older hospitalized patients. Eur. J. Clin. Nutr..

[45] Vanoh D., Shahar S., Yahya H.M., Din N.C., Ludin A.F.M., Singh D.K.A., Sharif R., Rajab N.F. (2021). Dietary Supplement Intake and Its Association with Cognitive Function, Physical Fitness, Depressive Symptoms, Nutritional Status and Biochemical Indices in a 3-Year Follow-Up Among Community Dwelling Older Adults: A Longitudinal Study. Clin. Interv. Aging.

